# Risk factors and prognostic factors associated with retinal detachment and visual outcomes in acute retinal necrosis

**DOI:** 10.1186/s12886-024-03533-3

**Published:** 2024-09-15

**Authors:** Yuxin Li, Li Chen, Pengcheng Li, Hao Kang, Yong Tao

**Affiliations:** 1 Department of Ophthalmology, Beijing Chaoyang Hospital, Capital Medical University, Gongren Tiyuchang Nanlu, Chaoyang District, Beijing, 100020 China; 2 Department of Ophthalmology, Union Hospital, Tongji Medical College, Huazhong University of Science and Technology, Beijing, Hubei China

**Keywords:** Acute retinal necrosis, Retinal detachment, Local antiviral therapy, Intraocular IL-8 concentration, Intraocular viral load

## Abstract

**Objective:**

To investigate the risk factors and prognostic factors that affect the long-term clinical outcomes of acute retinal necrosis (ARN).

**Methods:**

A retrospective study of patients with ARN who underwent treatment and completed follow-up in our ophthalmology department from 2011 to 2021 was conducted. The incidence and risk factors of retinal detachment (RD) and prognostic factors affecting long-term clinical outcomes, such as late-onset RD and final vision loss (< 20/200), were analyzed.

**Results:**

Totally 59 ARN patients (65 eyes) with an average follow-up of 48.9 months were enrolled. During the follow-up period, RD occurred in 34 eyes (52.3%). The risk factors for RD included quadrants of involved retinal necrosis (odds ratio [OR], 4.181; 95% confidence interval [CI], 1.950–10.834) and initial intraocular viral load (OR, 1.721; 95% CI, 1.071–3.083). Early intravitreal antiviral treatment (OR, 1.204; 95% CI, 1.040–1.480) was independently associated with a decreased risk of late-onset RD. The factors independently associated with an increased risk of final vision loss were worse initial visual acuity (OR, 3.895; 95% CI, 1.551–13.662) and late-onset RD (OR, 8.043; 95% CI, 1.380–67.216). In addition, we utilized the fluctuating magnitude of viral load to quantify the extent of its reduction in comparison to its original value following the initial intravitreal antiviral injection (IAI). This ratio was strongly related to initial intraocular IL-8 concentration (Spearman correlation coefficient=-0.741, *P* = 0.000) and moderately related to the initial degree of aqueous flare (Spearman correlation coefficient=-0.508, *P* = 0.010).

**Conclusion:**

RD is a common and severe complication of ARN with multiple risk factors, such as initial retinitis involvement area and initial intraocular viral load. Active local antiviral therapy may reduce the risk of late-onset RD. The antiviral medication should be adjusted according to the inflammatory state. Therefore, timely detection of causative viruses and intensive systemic and local antiviral therapy is crucial for preserving visual function in ARN patients.

## Introduction

Acute retinal necrosis (ARN) is an inflammatory ocular disease primarily caused by viral infection, typically characterized by retinal necrosis, retinitis predominantly of retinal arteries, moderate to severe vitreous opacity, and retinal detachment (RD). ARN is mainly caused by herpes simplex virus (HSV) and varicella-zoster virus (VZV) [1, 2]. ARN has a rapid onset and progression, leading to misdiagnosis or missed diagnosis. If not treated timely and adequately, ARN may result in vision loss and phthisis [3].

RD is the most common complication of ARN, which can cause further vision loss and poor visual prognosis in ARN patients [4]. Factors that may influence the occurrence of RD and final visual outcomes in ARN patients include the type of causative virus, extent of retinal necrosis, intraocular inflammatory responses including the range of retinitis and vitreous fluid dynamics, systemic immune status, and factors related to delays or inadequacies in treatment [5]. These factors must be identified and evaluated to guide clinical decision-making and improve treatment outcomes. Detecting viral nucleic acid in the intraocular fluid by polymerase chain reaction (PCR) technology can provide substantial evidence for diagnosing ARN syndrome by qualitatively confirming the viral type, quantitatively observing disease progression, and evaluating treatment effects [6, 7]. Some research results indicate that aqueous inflammatory cytokines such as IL-8 can reflect the inflammatory state of ARN syndrome [8]. However, there are few studies on the impact of initial viral load and inflammatory cytokine levels on ARN’s final vision outcomes and secondary RD occurrence. Moreover, since the inflammatory response caused by ARN often involves both the anterior and posterior segments of the eye simultaneously, it is necessary to investigate the impact of initial anterior uveitis-related signs such as keratic precipitate (KP), anterior chamber floating cells and aqueous flare on the clinical outcomes in ARN patients.

The treatment of ARN is challenging and controversial. The main goals of treatment are to control viral infection supplemented by systemic anti-inflammatory and antithrombotic therapy and prevent or repair RD by surgical methods such as pars plana vitrectomy (PPV) or prophylactic laser photocoagulation [9, 10]. System antiviral therapy is the primary non-surgical treatment method [11] Intravenous and oral antiviral therapies are widely recognized as the standard treatment modalities for ARN, demonstrating substantial efficacy in mitigating retinal inflammation and preventing involvement of the contralateral eye [12]. Intravitreal injection of antiviral drugs, such as foscarnet or ganciclovir, can provide additional support [13]. However, current studies have reported mixed effects of intravitreal antiviral injection (IAI) as an adjunctive to systemic antiviral therapy. Some researchers advocate using IAI because it may increase the intraocular concentration of antiviral drugs, especially in eyes with occlusive vasculitis, and inhibit the penetration of systemic antiviral drugs to reduce systemic adverse reactions [14]. Others have not found significant benefits or higher RD incidence. More importantly, there is no consensus on the optimal timing of intravitreal antiviral therapy [15].

Despite various systemic and local treatment methods for ARN, the risk of RD and final vision loss remains challenging. Most previous studies relied on case series or small cohorts of ARN patients with uncontrolled heterogeneous characteristics, and selection bias may considerably impact the results [16]. Therefore, we conducted this relatively sizeable retrospective study with demographic, clinical, and treatment data of ARN patients to investigate the long-term outcomes of ARN. We aim to evaluate the risk factors for RD in ARN patients and the effects of various prognosis factors on late-onset RD and final vision loss in ARN patients. We hope our findings provide evidence for the clinical management of ARN and the optimal timing of local antiviral therapy.

## Methods

### Study design and subjects

In this retrospective study, we reviewed the electronic medical records of ARN patients who received treatment and follow-up at Beijing Chaoyang Hospital Affiliated to Capital Medical University from September 2011 to September 2021. The diagnosis of ARN was based on the criteria of the American Uveitis Society guidelines [17]. The diagnostic tools employed included typical ocular manifestations of the patients and presentations of viral infection outside the eye, invasive diagnostic methods, and laboratory examinations such as PCR testing of aqueous or vitreous humor for viral detection, intraocular fluid protein analysis, fluorescein angiography, ultrasound biomicroscopy (UBM), optical coherence tomography (OCT), and ultrasonography of the eye [18]. We excluded all patients who were not followed for at least six months. This study was performed in accordance with the Declaration of Helsinki and was approved by the Ethics Committee of Beijing Chaoyang Hospital Affiliated to Capital Medical University (No. 2018-4-3-3). All patients provided written informed consent.

### Data collection

We extracted the following data from the electronic medical record system: demographic characteristics (age, gender, and follow-up period), immune status (immunocompetent or immunosuppressed), causative virus type, and ophthalmic data, including clinical features of ARN, as well as the drugs and surgical therapies used. We used a standardized data entry form to collect and manage data and performed regular quality checks and cleaning. We also verified or corrected missing or abnormal values by contacting the original data sources as much as possible. Genomic DNA of the causative virus was measured using the virus nucleic acid assay kit (Liferiver, Shanghai), and PCR was performed to detect the intraocular fluid viral load using Multicolor Real-time PCR Detection System (LineGene 9600 Plus, BIOER TECHNOLOGY, Hangzhou). We used the CBA Flexset kit to detect the IL-8 content in aqueous humor on the BD flow cytometer.

The ophthalmic data collected from the medical records included intraocular pressure (IOP) and visual acuity (VA) results (baseline and final follow-up visual acuity). International standard visual acuity charts were used for testing and converted to the logarithm of the minimum resolution angle (logMAR) to measure visual acuity. For a visual acuity below 20/2000, the following logMAR conversions were used: counting fingers = 2.3; hand motion = 2.6; light perception = 2.9; no light perception = 3.1 [19]. We assumed a specific feature was absent if the patient’s electronic medical record did not contain information. The ARN clinical features collected by two retina specialists based on ophthalmic examination were as follows: KP (presence or absence), degree of aqueous flare (graded as 0,1,2,3 by senior clinician grading), degree of anterior chamber floating cells (graded as 0, 1+, 2+, 3+, and 4 + using the grading system proposed by the Standardization of Uveitis Nomenclature [SUN] Working Group), involvement of optic nerve (optic nerve edema, atrophy or hemorrhage), and number of involved retinal quadrants of necrotizing retinitis involved retinal quadrant (graded as 1,<25%; 2,25-50%; 3,50-75%; 4,>75%) [19, 20].

All ARN patients initially received systemic therapy and IAI based on the patient’s viral load and causative virus type. We also applied standard adjunctive therapies, such as prophylactic laser photocoagulation, systemic corticosteroids, and aspirin, in eyes where the area of retinal necrosis expanded, vitreous opacity worsened after systemic antiviral therapy, or retinitis involved the optic nerve or macula. Surgery involving 25-gauge PPV, extensive prophylactic endolaser treatment, and silicone oil tamponade is typically indicated for patients with RD or severe vitritis that does not respond to systemic and intravitreal antiviral therapy.

RD was divided into two categories for patients with long-term follow-up: RD at initial presentation of ARN and late-onset RD. Late-onset RD was defined as RD that occurred three months after ARN diagnosis or after surgery due to RD at the initial presentation of ARN. The final visual outcome was divided into two groups according to the World Health Organization Criteria for severe visual impairment, which was defined in this study as vision loss: 1.0 logMAR or better (≥ 20/200) and worse than 1.0 logMAR (< 20/200) [21]. 

### Statistical methods

We performed descriptive statistics to analyze the risk factors for RD, the prognostic factors for late-onset RD, and final visual loss in ARN patients. We used the generalized estimating equation for logistic regression to account for the correlation between bilateral cases. We performed univariate and multivariate analyses using binary logistic regression to ascertain the effects of selected clinical or therapeutic factors. We included variables with P values < 0.2 in univariate analysis in multivariate analysis and calculated the corresponding odds ratios (OR) and 95% confidence intervals (CI). We also plotted the receiver operating characteristic (ROC) curves for the difference significance variables and computed the area under the curve (AUC) to measure the diagnostic accuracy of the variables. We selected the optimal cutoff value for each variable based on the maximum value of Youden’s index, defined as the sum of sensitivity and specificity. Spearman correlation analysis explored the relationship between the change of early viral load after IAI and initial inflammatory indicators, including anterior uveitis-related signs and IL-8 content. For continuous variables that followed a normal distribution, we used mean ± standard deviation to describe them; for continuous variables that did not follow the normal distribution, we used median and interquartile range to describe them. We set *P* < 0.05 as the significance level. Statistical analysis was performed using SPSS v24.0 (SPSS Inc., Armonk, NY, USA) and the RStudio (Windows desktop, version 4.0.2 software).

## Results

### Demographic and essential clinical characteristics of patients

The demographic information and basic clinical features of the patients are summarized in Table 1. Sixty-eight patients (75 eyes) were diagnosed with ARN. Seven patients (8 eyes) were excluded because they either refused treatment or did not complete the 6-month follow-up. Two patients (2 eyes) with ophthalmic comorbidities, such as choroidal retinitis or ocular trauma history, were also excluded.

Therefore, the analysis included 59 patients (male:39; female:20) with 65 eyes. The mean age at diagnosis by eye was 46.71 ± 14.52 (12–70 years old). The mean follow-up period was 48.88 ± 25.79 months (6-108 months). Five patients (8.5%) had immunodeficiency, including post-bone marrow transplantation (2 cases) and AIDS (3 cases). According to the aqueous or vitreous PCR results, the 65 eyes’ viral etiology included VZV in 61 eyes (93.8%) and HSV in 4 eyes (6.2%). The median and interquartile range of initial viral load before treatment was 2.69 × 10^5^ (1.86 × 10^3^, 6.16 × 10^5^) copies/ml, and the initial IL8 content was 121.40 (57.60, 398.70) pg/ml. During the treatment and follow-up period, RD occurred in 34 eyes (52.3%), of which ten eyes were late-onset RD.

**Table 1:** Demographic characteristics and basic clinical data of patients with acute retinal necrosis

Characteristic	Total^†^
**No. of patients**	59
**Eyes affected**	65
**Age at diagnosis by eye, years**	46.71 ±14.52
**Gender, male**	39/59 (66.1%)
**Follow-up period, months**	48.88 ±25.79
**Immunosuppressed status**	5/59 (8.5%)
**IOP, mmHg**	14.00 (11.50, 17.20)
**Initial visual acuity (logMAR)**	0.90 (0.63, 1.33)
**Viral etiology**	
HSV	4/65 (6.2%)
VZV	61/65 (93.8%)
**Viral load (copies/ml)**	2.69 × 10^5^ (1.86 × 10^3^, 6.16 × 10^5^)
**IL-8 content (pg/ml)**	121.40 (57.60, 398.70)

^**†**^Mean ± SD; n / total (percent of total); Median (interquartile range)

### Treatment management

All patients received systemic antiviral therapy and IAI. Our protocol for systemic antiviral therapy is as follows: Acyclovir is administered at a dosage of 10–15 mg/kg intravenously over 1 h, three times a day. This intravenous medication is continued for 10 days to 3 weeks, followed by oral medication. The oral dosing regimen is 400–800 mg five times a day for 4–6 weeks. Alternative medications such as ganciclovir and foscarnet are considered in cases where acyclovir treatment proves to be ineffective. Local IAI medications include ganciclovir or foscarnet, with an average of 3 [2, 5] injections. The average interval from symptom onset to primary IAI is 20 [15, 22] days. After starting antiviral therapy, 15 patients (25.9%) received oral corticosteroid therapy, 43 patients (66.2%) received oral aspirin therapy, and four patients (6.9%) received prophylactic laser therapy. PPV was performed in all 34 eyes with RD and one with severe vitritis before RD.

### Treatment outcomes: RD and VA

Within three months after the initial diagnosis of ARN, 31 eyes developed RD and required PPV. Seven eyes had recurrent RD after surgery. For the remaining 34 eyes that showed no signs of RD at presentation, three eyes developed late-onset RD at least three months after the diagnosis of ARN. Therefore, a total of 10 eyes developed late-onset RD. The median and interquartile range of final follow-up VA was 1.2 (0.5,2.6) logMAR, which was significantly lower than the initial VA of 0.9 (0.6,1.1) logMAR (*P* < 0.05). Final vision loss was observed in 35 eyes (53.8%). In the early-onset RD group, the final follow-up VA was 2.0 (1.1, 2.6) logMAR, which was significantly worse than the initial VA of 1.5 (0.9, 2.5) logMAR (*P* < 0.05). In the late-onset RD group, the final follow-up VA was 2.6 (1.1, 2.9) logMAR, which was significantly worse than the initial VA of 1.0 (0.8, 2.7) logMAR (*P* < 0.05).

### Risk factors for RD

Demographic and clinical characteristics were evaluated by RD (Table 2). We assessed the risk factors for RD by univariate and multivariate analysis (Table 3). Compared with eyes that did not experience RD with a mean VA of 0.75 (0.53, 1.08) logMAR, eyes that experienced RD during follow-up had significantly worse mean VA of 1.00 (0.83, 2.00) logMAR at presentation (*P* < 0.05). Eyes that experienced RD had necrotizing retinitis involving more than 75% of quadrants in 27 eyes (79.4%), which was significantly higher than eyes that did not experience RD in 6 eyes (19.4%) (*P* < 0.001). Eighteen (52.9%) eyes that experienced RD had anterior chamber flare levels greater than or equal to grade 2, which was significantly higher than eight eyes (25.9%) that did not experience RD (*P* < 0.05). Eyes that experienced RD had a mean aqueous or vitreous log10(viral load) of 6.60 (5.83, 7.16), which was significantly higher than eyes that did not experience RD with a mean of 5.24 (3.93, 5.99) (*P* < 0.05). The median and interquartile range of IL-8 content before treatment in RD patients was 826.39 (376.62, 3,939.50) pg/ml, which was higher than 428.25 (199.53, 1,191.92) in no RD-developed eyes. However, the difference was not statistically significant. We created a multivariate logistic regression model using the variables related with RD (*P* < 0.20) in the univariate analysis (Table 3). In this model, the OR value for the more considerable extent of necrotizing retinitis was 4.181 (95% CI, 1.950-10.834) (*P* < 0.001), and the OR value for the enormous viral load was 1.721 (95% CI, 1.07–3.083) (*P* < 0.05).

**Table 2:** Clinical characteristics related with retinal detachment in eyes with acute retinal necrosis

Characteristic	No Retinal Detachment Developed^†^	Retinal Detachment Developed^†^
**Age at diagnosis by eye, years**	45.00 ± 17.46	48.44 ± 13.04
**Gender, male**	20/31 (64.5%)	25/34 (73.5%)
**Viral etiology**		
HSV	2/31 (6.5%)	2/34 (5.9%)
VZV	29/31 (93.5%)	32/34 (94.1%)
**Optic nerve involvement**	0/31 (0.0%)	3/34 (8.8%)
**IOP, mmHg**	14.00 (11.50, 17.20)	13.50 (11.25, 16.75)
**Keratic precipitates**	28/31 (90.3%)	29/34 (85.3%)
**Involved retinal quadrants of necrotizing retinitis**		
1 (<25%)	8/31 (25.8%)	0/34 (0.0%)
2 (25-50%)	11/31 (35.5%)	2/34 (5.9%)
3 (50-75%)	6/31 (19.4%)	5/34 (14.7%)
4 (>75%)	6/31 (19.4%)	27/34 (79.4%)
**Immunosuppressed**	5/31 (16.1%)	2/34 (5.9%)
**Initial visual acuity (logMAR)**	0.75 (0.53, 1.08)	1.00 (0.83, 2.00)
**Degree of anterior chamber cells**		
0	13/29 (44.8%)	7/34 (20.6%)
1	9/29 (31.0%)	13/34 (38.2%)
2	3/29 (10.3%)	8/34 (23.5%)
3	1/29 (3.4%)	5/34 (14.7%)
4	3/29 (10.3%)	1/34 (2.9%)
**Degree of aqueous flare**		
0	11/31 (35.5%)	3/34 (8.8%)
1	12/31 (38.7%)	13/34 (38.2%)
2	6/31 (19.4%)	15/34 (44.1%)
3	2/31 (6.5%)	3/34 (8.8%)
**Viral load** ^‡^	5.24 (3.93, 5.99)	6.60 (5.83, 7.16)
**IL-8 content (pg/ml)**	428.25 (199.53, 1,191.92)	826.39 (376.62, 3,939.50)

^**†**^Mean ± SD; n / total (percent of total); Median (interquartile range)

^‡^The viral load is expressed by log10 (viral load, copies/ml)

**Table 3:** Univariate and Multivariate regression analysis for eyes with acute retinal necrosis: estimation of risk factors for retinal detachment

	Univariate Analysis			Multivariate Analysis		
Characteristic	OR^†^	95% CI^†^	*p*-value	OR^†^	95% CI^†^	*p*-value
**Age at diagnosis by eye, years**	1.015	0.983, 1.050	0.363			
**Gender, male**	0.655	0.222, 1.884	0.433			
**Viral etiology**	1.103	0.126, 9.682	0.924			
**IOP, mmHg**	0.952	0.853, 1.056	0.354			
**Keratic precipitates**	0.621	0.118, 2.775	0.540			
**Involved retinal quadrants of necrotizing retinitis**	5.549	2.753, 13.637	**<0.001** ^***^	4.181	1.950, 10.834	**<0.001** ^***^
**Immunosuppressed**	0.323	0.044, 1.636	0.198			
**Initial visual acuity (logMAR)**	2.350	1.183, 5.398	**0.024** ^*^			
**Degree of anterior chamber cells**	1.325	0.863, 2.112	0.211			
**Degree of aqueous flare**	2.191	1.210, 4.273	**0.014** ^*^			
**Viral load** ^‡^	2.003	1.300, 3.440	**0.004** ^**^	1.721	1.071, 3.083	**0.038** ^*^
**IL-8 content (pg/ml)**	1.000	1.000, 1.000	0.389			

^†^OR = Odds Ratio, CI = Confidence Interval

^‡^The viral load is expressed by log10 (viral load, copies/ml).

*P < 0.05，**P < 0.01，***P < 0.001

### Prognostic factors associated with late-onset RD

**Fig. 1:**
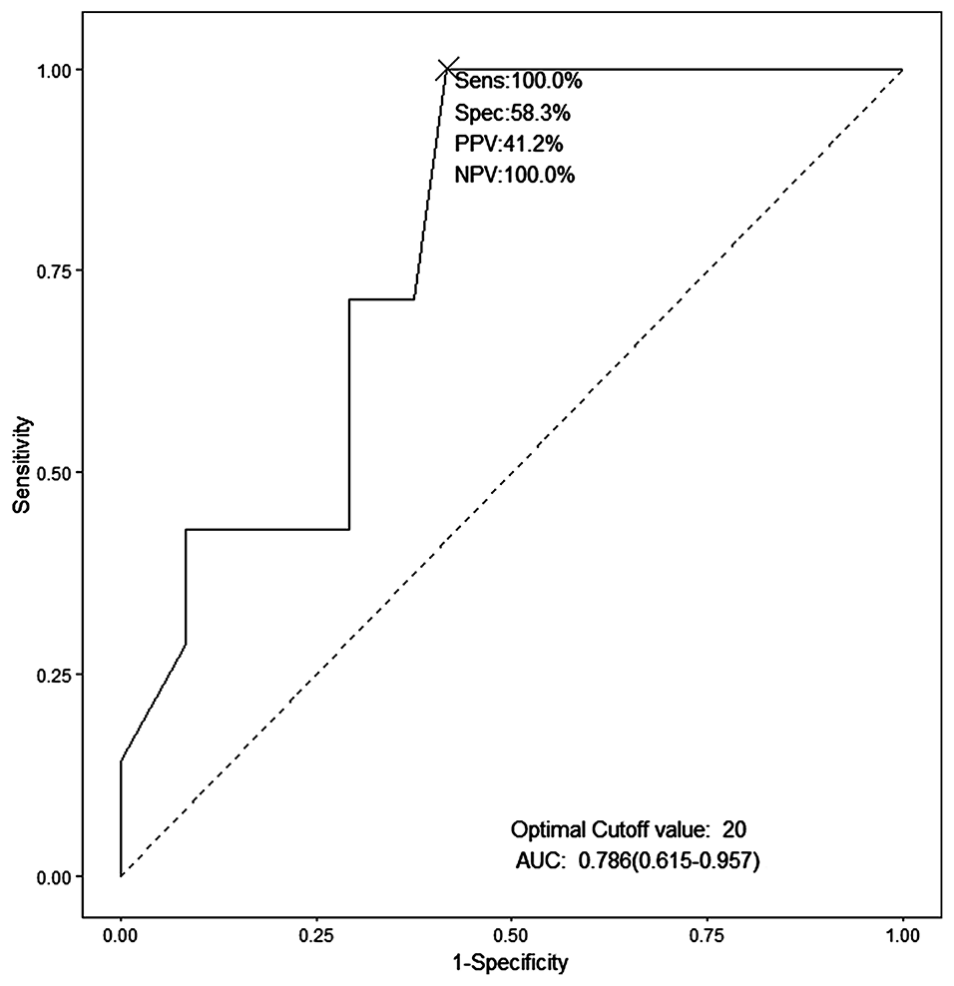
Receiver operating characteristic (ROC) curve of the interval between ARN symptoms appear and primary IAI for late-onset RDeristic (ROC) curve of Initial visual acuity (logMAR) for final visual loss. AUC: area under the curve; sens: sensitivity; spec: specificity; PPV: positive predictive value; NPV: negative predictive value

We grouped the cohort according to late-onset RD occurrence and compared the groups’ baseline characteristics and treatment factors (Table 4). We assessed the prognostic factors for late-onset RD by univariate and multivariate analysis (Table 5). Late-onset RD eyes had a worse initial VA of 0.95 (0.80, 2.45) logMAR (while the initial VA of no late-onset RD developed eyes was 0.90 (0.60, 1.10) logMAR). Moreover, all late-onset RD involved more than 75% retinal quadrants of necrotizing retinitis at presentation. In the multivariate model, only the interval between symptom onset and primary IAI (OR, 1.204; 95% CI, 1.040–1.480; *P* < 0.05) showed a statistically significant increased risk of late-onset RD. We performed ROC curve analysis with the interval between symptom onset and primary IAI as the test variable and late-onset RD as the status variable to evaluate its predictive performance. The model AUC value was 0.786 (0.615–0.957), the optimal cutoff value was 20 days, the sensitivity was 100%, the specificity was 58.3%, the positive predictive value (PPV) was 41.2%, the negative predictive value (NPV) was 100% (Fig. 1).

**Table 4:** Basic clinical data and treatment factors related with late-onset retinal detachment in eyes with acute retinal necrosis

Characteristic	No Late-onset Retinal Detachment Developed^†^	Late-onset Retinal Detachment Developed^†^
**Age at diagnosis by eye, years**	46.15 ± 16.01	50.40 ± 10.27
**Gender, male**	38/55 (69.1%)	7/10 (70.0%)
**Viral etiology**		
HSV	3/55 (5.5%)	1/10 (10.0%)
VZV	52/55 (94.5%)	9/10 (90.0%)
**Optic nerve involvement**	2/55 (3.6%)	1/10 (10.0%)
**IOP, mmHg**	14.00 (11.10, 16.70)	14.00 (11.55, 18.18)
**Keratic precipitates**	49/55 (89.1%)	8/10 (80.0%)
**Involved retinal quadrants of necrotizing retinitis**		
1 (<25%)	8/55 (14.5%)	0/10 (0.0%)
2 (25-50%)	13/55 (23.6%)	0/10 (0.0%)
3 (50-75%)	11/55 (20.0%)	0/10 (0.0%)
4 (>75%)	23/55 (41.8%)	10/10 (100.0%)
**Immunosuppressed**	6/55 (10.9%)	1/10 (10.0%)
**Initial visual acuity (logMAR)**	0.90 (0.60, 1.10)	0.95 (0.80, 2.45)
**Degree of anterior chamber cells**		
0	18/53 (34.0%)	2/10 (20.0%)
1	19/53 (35.8%)	3/10 (30.0%)
2	7/53 (13.2%)	4/10 (40.0%)
3	5/53 (9.4%)	1/10 (10.0%)
4	4/53 (7.5%)	0/10 (0.0%)
**Degree of aqueous flare**		
0	12/55 (21.8%)	2/10 (20.0%)
1	21/55 (38.2%)	4/10 (40.0%)
2	17/55 (30.9%)	4/10 (40.0%)
3	5/55 (9.1%)	0/10 (0.0%)
**Viral load** ^‡^	5.77 (4.56, 6.65)	6.95 (6.38, 7.31)
**lL-8 content (pg/ml)**	485.50 (218.23, 2,312.93)	694.90 (617.80, 772.00)
**Oral steroid administration**	11/55 (20.0%)	4/10 (40.0%)
**Oral aspirin administration**	38/55 (69.1%)	8/10 (80.0%)
**The interval between ARN symptom onset and primary IAI, days** ^**§**^	17.50 (13.75, 25.00)	24.00 (21.50, 29.00)
**Times of IAI** ^**§**^	3.00 (2.00, 5.00)	2.00 (1.00, 3.75)
**Prophylactic laser administration**	2/55 (3.6%)	1/10 (10.0%)

^†^Mean ± SD; n / total (percent of total); Median (interquartile range)

^‡^The viral load is expressed by log10 (viral load, copies/ml)

^§^IAI: intravitreal antiviral injection

**Table 5:** Univariate and Multivariate regression analysis for eyes with acute retinal necrosis: estimation of prognostic factors associated with late-onset retinal detachment

	Univariate Analysis			Multivariate Analysis		
Characteristic	OR^†^	95% CI^†^	*p*-value	OR^†^	95% CI^†^	*p*-value
**Age at diagnosis by eye, years**	1.02	0.975, 1.072	0.418			
**Gender, male**	0.958	0.189, 3.918	0.954			
**Viral etiology**	0.519	0.059, 11.138	0.588			
**Optic nerve involvement**	2.944	0.129, 34.043	0.398			
**IOP, mmHg**	0.998	0.854, 1.145	0.973			
**Keratic precipitates**	0.49	0.092, 3.735	0.428			
**Immunosuppressed**	0.87	0.043, 6.012	0.903			
**Initial visual acuity (logMAR)**	1.787	0.812, 3.866	0.136			
**Degree of anterior chamber cells**	1.143	0.634, 1.970	0.637			
**Degree of aqueous flare**	0.91	0.410, 1.961	0.811			
**Viral load** ^‡^	2.01	0.895, 6.441	0.155			
**IL-8 content (pg/ml)**	1	0.997, 1.000	0.633			
**Oral steroid administration**	2.836	0.613, 12.615	0.166			
**Oral aspirin administration**	1.789	0.396, 12.695	0.49			
**The interval between symptom onset and primary IAI, days** ^**§**^	1.204	1.040, 1.480	0.033^*^	1.204	1.040, 1.480	0.033^*^
**Times of IAI** ^**§**^	0.673	0.401, 1.012	0.093			
**Prophylactic laser administration**	2.944	0.129, 34.043	0.398			

^**†**^OR = Odds Ratio, CI = Confidence Interval

^‡^The viral load is expressed by log10 (viral load, copies/ml)

^§^IAI: intravitreal antiviral injection

*P < 0.05，**P < 0.01，***P < 0.001

### Prognostic factors associated with final vision loss

**Fig. 2:**
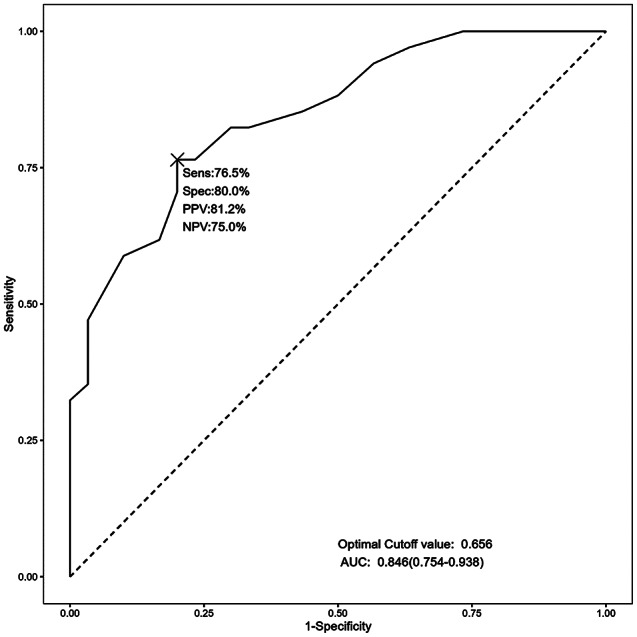
Receiver operating characteristic (ROC) curve of Initial visual acuity (logMAR) for final visual loss. AUC: area under the curve; sens: sensitivity; spec: specificity; PPV: positive predictive value; NPV: negative predictive value

We also grouped the cohort according to the final vision outcome and compared the groups’ baseline characteristics and treatment factors (Table 6). We assessed the prognostic factors for final vision loss by univariate and multivariate analysis (Table 7). Lower initial VA (the no final vision loss group had 0.70 (0.53, 1.00) logMAR, while the final vision loss group had 1.00 (0.90, 2.53) loMAR, *P* = 0.001), RD at initial presentation of ARN (6.7% and 22.9%, respectively, *p* = 0.011) and late-onset RD (*P* = 0.089) increased the risk of final vision loss. Regarding treatment factors, no clear correlation was found between treatment modality and final vision loss. The multivariate logistic regression model confirmed that worse initial VA (OR, 3.895; 95% CI, 1.551–13.662; *P* = 0.013), RD at initial presentation of ARN (OR, 10.84; 95% CI, 1.62–72.41; *p* = 0.014) and late-onset RD (OR, 6.735; 95% CI, 1.876–27.282; *P* = 0.005) were statistically significant factors on final vision loss. We performed ROC curve analysis with initial VA as the test variable and final vision loss as the outcome variable to evaluate its predictive performance. The model AUC value was 0.846 (0.754, 0.938), the optimal Cutoff value was 0.656 logMAR; sensitivity was 76.5%, specificity was 80.0%, PPV was 81.2%, and NPV was 75.0% (Fig. 2).

**Table 6:** Basic clinical data and treatment factors related with final vision outcomes in eyes with acute retinal necrosis

Characteristic	Final visual acuity≥20/200^†^	Final visual acuity <20/200^†^
**Age at diagnosis by eye, years**	43.20 ± 16.29	49.89 ± 13.86
**Gender, male**	19/30 (63.3%)	26/35 (74.3%)
**Viral etiology**		
HSV	2/30 (6.7%)	2/35 (5.7%)
VZV	28/30 (93.3%)	33/35 (94.3%)
**Optic nerve involvement**	1/30 (3.3%)	2/35 (5.7%)
**IOP, mmHg**	15.71 ± 5.50	13.88 ± 3.87
**Keratic precipitates**	27/30 (90.0%)	30/35 (85.7%)
**Involved retinal quadrants of necrotizing retinitis**		
1 (<25%)	8/30 (26.7%)	0/35 (0.0%)
2 (25-50%)	11/30 (36.7%)	2/35 (5.7%)
3 (50-75%)	3/30 (10.0%)	8/35 (22.9%)
4 (>75%)	8/30 (26.7%)	25/35 (71.4%)
**Immunosuppressed**	4/30 (13.3%)	3/35 (8.6%)
**Initial visual acuity (logMAR)**	0.70 (0.53, 1.00)	1.00 (0.90, 2.53)
**Degree of anterior chamber cells**		
0	10/28 (35.7%)	10/35 (28.6%)
1	8/28 (28.6%)	14/35 (40.0%)
2	4/28 (14.3%)	7/35 (20.0%)
3	3/28 (10.7%)	3/35 (8.6%)
4	3/28 (10.7%)	1/35 (2.9%)
**Degree of aqueous flare**		
0	8/30 (26.7%)	6/35 (17.1%)
1	12/30 (40.0%)	13/35 (37.1%)
2	6/30 (20.0%)	15/35 (42.9%)
3	4/30 (13.3%)	1/35 (2.9%)
**Viral load** ^‡^	5.43 (4.58, 6.56)	6.05 (5.04, 7.05)
**IL-8 content (pg/ml)**	529.85 (201.28, 1,248.14)	627.84 (345.80, 3,710.55)
**Oral steroid administration**	8/30 (26.7%)	7/35 (20.0%)
**Oral aspirin administration**	20/30 (66.7%)	26/35 (74.3%)
**The interval between symptom onset and primary IAI, days** ^**§**^	18.65 ± 6.42	20.71 ± 9.71
**Times of IAI** ^**§**^	3.00 (2.00, 4.00)	4.00 (2.00, 5.00)
**Prophylactic laser administration**	0/30 (0.0%)	3/35 (8.6%)
**Late-onset RD**	2/30 (6.7%)	8/35 (22.9%)
**RD at initial presentation of ARN**	6/30 (20.0%)	18/35 (51.4%)

^**†**^Mean ± SD; n / total (percent of total); Median (interquartile range)

^‡^The viral load is expressed by log10 (viral load, copies/ml)

^**§**^IAI: intravitreal antiviral injection

**Table 7:** Univariate and Multivariate regression analysis for eyes with acute retinal necrosis: estimation of prognostic factors associated with final vision loss (<20/200)

	Univariate Analysis			Multivariate Analysis		
Characteristic	OR^†^	95% CI^†^	*p*-value	OR^†^	95% CI^†^	*p*-value
**Age at diagnosis by eye, years**	1.030	0.997, 1.067	0.082			
**Gender, male**	0.598	0.203, 1.723	0.342			
**Viral etiology**	1.179	0.134, 10.344	0.874			
**Optic nerve involvement**	1.758	0.160, 38.979	0.652			
**IOP, mmHg**	0.918	0.818, 1.021	0.125			
**Keratic precipitates**	0.667	0.127, 2.979	0.602			
**Involved retinal quadrants of necrotizing retinitis**	4.063	2.207, 8.614	**<0.001** ^***^			
**Immunosuppressed**	0.605	0.111, 2.987	0.535			
**Initial visual acuity (logMAR)**	4.955	2.016, 17.903	**0.003** ^**^	3.895	1.551, 13.662	**0.011** ^*^
**Degree of anterior chamber cells**	1.159	0.665, 2.047	0.603			
**Degree of aqueous flare**	1.159	0.665, 2.047	0.603			
**Viral load** ^‡^	1.137	0.799, 1.641	0.477			
**IL-8 content (pg/ml)**	1.000	1.000, 1.000	0.404			
**Oral steroid administration**	0.799	0.241, 2.599	0.708			
**Oral aspirin administration**	1.444	0.493, 4.296	0.502			
**The interval between symptom onset and primary IAI, days** ^**§**^	1.035	0.945, 1.144	0.470			
**Times of IAI** ^**§**^	1.075	0.868, 1.377	0.522			
**Late-onset RD**	4.148	0.936, 29.171	0.089	8.043	1.380, 67.216	**0.029** ^*^
**RD at initial presentation of ARN**	4.235	1.450, 13.784	**0.011** ^*^	6.735	1.876, 27.282	**0.005** ^**^

^**†**^Mean ± SD; n / total (percent of total); Median (interquartile range)

^‡^The viral load is expressed by log10 (viral load, copies/ml)

^**§**^IAI: intravitreal antiviral injection

*P < 0.05，**P < 0.01，***P < 0.001

### Correlation of early viral load and initial inflammatory indicators

Table 8 shows the correlation between the early viral load and initial inflammatory indicators. We used the changing amplitude of viral load to measure how much it decreased relatively to its initial value after the first IAI. This ratio was strongly related to initial IL8 content (Spearman correlation coefficient=-0.741, *P* = 0.000) and moderately related to the initial degree of aqueous flare (Spearman correlation coefficient=-0.508, *P* = 0.010).

**Table 8:** Spearman correlation analysis between the early viral load and initial inflammatory indicators

	Initial viral load^†^		Viral load after first IAI^†^	Changing amplitude of viral load after first IAI^§^		
Variables	Correlation coefficient	*p*-value	Correlation coefficient	*p*-value	Correlation coefficient	*p*-value
**Degree of anterior chamber cells**	0.336	**0.018** ^*^	0.222	0.285	-0.206	0.323
**Degree of aqueous flare**	0.452	**0.001** ^**^	0.666	**0.000** ^***^	-0.508	**0.010** ^**^
**lL-8 content** ^‡^	0.673	**0.000** ^***^	0.890	**0.000** ^***^	-0.741	**0.000** ^***^

^**†**^The viral load is expressed by log10 (viral load, copies/ml)

^‡^The IL-8 content is expressed by log10 (IL-8 content, pg/ml)

^**§**^IAI: intravitreal antiviral injection; the changing amplitude of viral load after the first IAI is the ratio between {log10 (initial viral load)-log10 (viral load after first IAI)} and log10 (initial viral load)

*P < 0.05，**P < 0.01，***P < 0.001

## Discussion

In this large cohort of PCR-confirmed ARN patients, the incidence of RD was 52.3%, consistent with the reported RD rate ranging from 30 to 75% in the literature. Our data demonstrated that higher initial viral load was an independent risk factor for RD in ARN patients, consistent with most scholars’ argument that viral load was related to retinal detachment [23]. A higher initial viral load may cause larger necrotic lesions in the affected eye and a higher chance of retinal detachment, which suggests that it is essential to perform intraocular fluid viral load detection early, which provides a basis for the timing of antiviral therapy [24, 25]. Notably, IL-8 content before treatment in RD eyes was higher than in no RD-developed eyes, although we did not observe any statistical difference between these groups. IL8 may be more conducive to monitoring inflammatory states and treatment responses.

Our study’s initial aqueous flare level was related to RD occurrence. The incidence of RD increased by 4.181 times for every additional retinal quadrant involved, which was the independent risk factor. In our clinical experience, RD may occur shortly after acute anterior uveitis occurs for eyes with ARN but usually lags by weeks or even months, occurring in the late scarring stage. These anterior segment inflammatory indicators may be more helpful in evaluating the treatment effect of intraocular inflammation during follow-up. In contrast, early posterior segment inflammatory indicators are more conducivmoreovere to providing early warning for preventing RD.

Ten eyes (15.4%) developed late-onset RD during treatment and follow-up, less than previously reported 26.3% [15]. Each patient in our study received combined systemic and local antiviral therapy, partly explaining the lower rate of late-onset RD caused by actively using intravitreal antiviral drugs. Our results showed that the interval from symptom onset to primary IAI influenced late-onset RD. Therefore, it further proved that early IAI was very important. According to ROC curve analysis, a cutoff point of 20 days from symptom onset to IAI in ARN patients had high sensitivity and specificity, highlighting the importance of early viral load detection and early IAI. We provided strong evidence of the benefit of early IAI and may provide a simple but informative data point for clinical decision-making and patient counseling. Moreover, we found no evidence of a correlation between the number of IAI treatments and the occurrence of late-onset RD in patients. Compared with other RD studies, patients included in our study received regular long-term IAI and follow-up for more than six months, providing solid data support for the safety of IAI for preventing late-onset RD [22].

In our study, 53.8% of patients had a final vision loss, comparable to the previously reported 46–68% rates [16, 26]. RD was one of the most important prognostic factors for final vision loss. Previous studies also suggested that poor initial visual acuity might affect the final visual outcomes [27]. Our results supported this argument as well. However, we did not find any harmful or beneficial associations of other treatments (such as systemic anticoagulation, corticosteroids, or prophylactic laser) with the outcomes of ARN treatment.

Moreover, our data demonstrated that a higher changing amplitude of viral load after the first IAI was correlated with initial inflammatory indicators, such as initial IL8 content and degree of aqueous flare. In 2015, the Japanese ARN diagnostic criteria emphasized the importance of anterior chamber inflammation indicators for diagnosing ARN [28]. This suggests that the level of inflammatory indicators could help assess the severity of intraocular inflammation, which would play an essential role in diagnosing and treating ARN. The low drug concentration caused by the accelerated aqueous humor circulation induced by the inflammatory state might result in a slight early viral load decline or increase [29]. Hence, the clinical antiviral medication and mode should be adjusted according to the inflammatory state.

This study has limitations such as its retrospective nature, variable follow-up intervals, and the referral nature of our practice, which may have biased our cohort towards eyes with more severe disease and higher RD risk. Therefore, prospective studies are needed to develop rigorous ARN assessment criteria further to improve the characterization of RD risk factors, early intervention of ARN, and best practices for patient counseling and monitoring.

## Conclusion

This retrospective observational study included 65 eyes with ARN and followed them for an average of 48.9 months. The results showed that the initial viral load and the early extent of retinitis were associated with the risk of RD. These factors may reflect the severity and progression of the infection, which can lead to retinal necrosis and detachment. Our study also showed that late-onset RD may increase the risk of final vision loss related to other factors, such as low initial vision. Therefore, timely detection of viral type and aggressive systemic and local antiviral therapy are crucial for preserving visual function. The shorter interval of IAI application was associated with a reduced risk of late-onset RD, which suggests that IAI can effectively suppress viral replication and inflammation in the vitreous cavity and prevent further damage to the retina. Therefore, eyes with low initial vision and more extensive retinitis or initial viral load at high risk of RD may require closer monitoring for final vision loss. In addition, early combined systemic and local antiviral therapy in these high-risk patients may reduce the risk of late-onset RD and final vision loss.

## Data Availability

The data that support the finding is available from the corresponding author on reasonable request.
